# Alternative activation of macrophages by filarial nematodes is MyD88-independent

**DOI:** 10.1016/j.imbio.2012.07.006

**Published:** 2013-04

**Authors:** Katie J. Mylonas, Marieke A. Hoeve, Andrew S. MacDonald, Judith E. Allen

**Affiliations:** a Institute of Immunology and Infection Research, Centre for Immunity, Infection & Evolution, School of Biological Sciences, The University of Edinburgh, West Mains Road, Edinburgh EH9 3JT, United Kingdom; b The Queen's Medical Research Institute, 47 Little France Crescent, Edinburgh EH16 4TJ, United Kingdom; c MRC Centre for Regenerative Medicine, University of Edinburgh, 49 Little France Crescent, Edinburgh EH16 4SB, United Kingdom

**Keywords:** MyD88, TLR, Macrophage, Th2, Filariasis

## Abstract

Alternative macrophage activation is largely defined by IL-4Rα stimulation but the contribution of Toll-like receptor (TLR) signaling to this phenotype is not currently known. We have investigated macrophage activation status under Th2 conditions in the absence of the core TLR adaptor molecule, MyD88. No impairment was observed in the ability of MyD88-deficient bone marrow derived macrophages to produce or express alternative activation markers, including arginase, *RELM-α* or *Ym1*, in response to IL-4 treatment *in vitro*. Further, we observed no difference in the ability of peritoneal exudate cells from nematode implanted wild type (WT) or MyD88-deficient mice to produce arginase or express the alternative activation markers RELM-α or Ym1. Therefore, MyD88 is not a fundamental requirement for Th2-driven macrophage alternative activation, either *in vitro* or *in vivo*.

## Introduction

Pattern recognition receptors (PRRs) recognize molecules that are broadly shared amongst pathogens and include the C-type lectin receptors, NOD-like receptors and Toll-like receptors (TLRs). TLRs expressed on antigen presenting cells such as macrophages play a central role in the activation of innate and adaptive immune responses (Iwasaki and Medzhitov 2004; Kawai and Akira 2011). Myeloid Differentiation Factor 88 (MyD88) is a critical adaptor molecule shared by many TLRs and signaling through most of these receptors is completely dependent on MyD88. However, MyD88-independent pathways also exist for some TLRs, *e.g.* TLR4 (Akira and Hoshino 2003).

In the absence of MyD88, Th1 responses are greatly diminished, revealing a key role for TLRs and MyD88-dependent signaling in the control of adaptive Th1 immunity (Adachi et al. 1998). Th2 responses, on the other hand, have been shown in some studies to be intact or even augmented in the absence of MyD88 (Schnare et al. 2001; Kaisho et al. 2002; Muraille et al. 2003; Chen et al. 2010; Gaddis et al. 2011), suggesting that Th2 induction does not require MyD88. However, other work indicates that TLR signaling can play a role in promoting Th2 responses. For example, Eisenbarth et al. (2002) found that low levels of LPS-induced signaling through TLR4 is necessary to induce Th2 responses to inhaled antigens in a mouse model of allergic sensitisation. Th2 induction was later found to be MyD88-dependent but this was reliant on the initial route of antigen exposure (Piggott et al. 2005). Therefore, whether signaling through MyD88 is required for a Th2 response may depend on the particular model under investigation. Beyond initiation of immunity, MyD88 can regulate antigen presenting cell effector function. Recognition of microbial products through TLRs, along with interferon-γ (IFN-γ) exposure, is known to polarize macrophages towards a classical activation state, defined by the production of antimicrobial products and pro-inflammatory mediators (Dalton et al. 1993; Aderem and Ulevitch 2000). In contrast, macrophages found in Th2 settings such as helminth infection, have been described as alternatively activated (Gordon and Martinez 2010), and in mice display IL-4/IL-13-dependent features, such as the expression of Arginase1, RELM-α and Ym1, as well as the ability to suppress the proliferation of neighbouring cells *ex vivo* (Loke et al. 2000, 2002; Mylonas et al. 2009; Jenkins and Allen 2010). However, the contribution of MyD88 to alternative macrophage activation is currently unclear.

Inflammatory pathology associated with filarial nematode infection can lead to lymphedema and elephantiasis (lymphatic filariasis) and ocular and skin damage (onchocerciasis) (Hoerauf et al. 2011; Babu et al. 2011). It was proposed originally that pathology may relate to LPS activity from *Wolbachia*, the endosymbiotic bacteria contained within filarial nematodes, acting through TLR-4 to cause production of the key pro-inflammatory cytokines IL-1β and TNF-α by macrophages (Taylor et al. 2000). However, *Wolbachia* was subsequently found to contain no LPS and fails to signal through TLR-4 (Hise et al. 2007). The pro-inflammatory activity of *Wolbachia* has more recently been attributed to MyD88-dependent TLR-2 and TLR-6 signals (Hise et al. 2007), although the ligands are not known. Filarial *Wolbachia* has been further implicated in T helper cell polarisation (Turner et al. 2009) but, for the most part, these studies have been performed *in vitro* or have utilized parasite extracts. An investigation into a role for MyD88 in macrophage activation or Th2 immunity during exposure to live infection is lacking.

In light of this, we have investigated whether MyD88 signaling impacts negatively or positively on macrophage phenotype or numbers using the wolbachia-containing filarial nematode, *Brugia malayi*. We first tested the *in vitro* capacity of MyD88 macrophages to become alternatively activated. Using wild type (WT) or MyD88^−/−^ bone marrow-derived macrophages (BMMφ) treated with IL-4, we found comparable levels of arginase activity and *Arginase1*, *RELM-α* and *Ym-1* mRNA expression. Somewhat more surprisingly, we also found no evidence for MyD88 involvement *in vivo*. Implantation of *B. malayi* adult worms into the peritoneal cavity is a potent Th2 stimulus that induces large numbers of alternatively activated macrophages (Loke et al. 2007). No significant differences between WT or MyD88^−/−^ mice were found in terms of cell recruitment profiles or alternative activation markers. In agreement with this, the Th2 cytokines induced following parasite implant were not altered. Together, this provides evidence that the adaptor protein MyD88 is not essential for Mφ alternative activation, either directly by IL-4 *in vitro*, or through exposure to a Th2 promoting parasitic helminth *in vivo*.

## Materials and methods

### Macrophage activation

BMM**φ** were prepared as described previously (Mylonas et al. 2009). Briefly, BM cells were seeded onto petri dishes at 7.5 × 10^6^ cells/plate and cultured in DMEM, supplemented with 25% foetal calf serum (FCS) (GIBCO), 25% L929 supernatant (as a source of M-CSF), 2 mM l-glutamine, 0.25 U/ml penicillin and 100 μg/ml streptomycin. The medium was replaced after four and six days and the macrophages collected on day 7. These BMM**φ** were transferred to 9 cm Petri dishes and left untreated in complete medium (DMEM, 10% FCS, 2 mM l-glutamine, 0.25 U/ml penicillin and 100 μg/ml streptomycin), or exposed for 18–24 h to recombinant IL-4 (20 ng/ml; BD Pharmingen). The BMM**φ** were then treated with LPS (100 ng/ml; *Escherichia coli* 0111:B4 Sigma–Aldrich) and IFN-γ (10 U/ml; BD Pharmingen) together or separately for a further 18–24 h.

### Mice and infection

All experiments used WT C57BL/6 or MyD88^−/−^ mice on a C57BL/6 background that were bred in house. Original MyD88^−/−^ breeders were generously provided by Prof. R. Grencis (University of Manchester) with the agreement of S. Akira (Osaka University). Mice were 6–8 weeks old at the start of the experiment and all animal work was conducted in accordance with the UK Animals (Scientific Prodecures) Act 1986. Adult *B. malayi* nematodes were removed from the peritoneal cavity of infected gerbils purchased from TRS Laboratories (Athens, GA) or maintained in house. Mice were surgically implanted intra-peritoneally (i.p.) with 5–6 live adult female nematodes. The peritoneal exudate cells (PECs) were harvested at peak cellular recruitment (Nair et al. 2005; Loke et al. 2007), 19d later, by thorough washing of the peritoneal cavity with 1 ml, and then a further 9 ml, ice-cold DMEM (Gibco). The first 1 ml lavage fluid was saved for protein analysis, and the cells from both lavage steps combined. As a control for non-Th2 polarised inflammation, mice were injected i.p. with 0.8 ml of 4% brewer modified thioglycollate medium (Becton Dickinson). Three days later, PECs were harvested as above. Recovered PECs were cultured in complete medium and the macrophages purified by adherence, as previously described (Nair et al. 2003).

### Flow cytometry

After blocking with 2% mouse serum cells were stained using the following mAb: F4/80-biotin, CD4-APC, CD8-PE, B220-PCP and SiglecF-PE, as well as the appropriate isotype control Abs. Samples were then acquired using BD LSRII, with data subsequently analysed by FlowJo (Tree Star, Inc.).

### Cytocentrifuge preparations

Cytocentrifuge preparations of 8 × 10^5^ cells in complete medium were made using a Shandon Cytospin. Slides were air-dried overnight and fixed for 10 min in cold methanol, followed by staining with Diff-Quik (Dade) according to the manufacturer's instructions. The cell populations were determined by microscopic examination (40× objective) of at least 100 cells per slide.

### Proliferation assay

Macrophages purified by adherence were co-cultured (1 × 10^5^ cells/well) in 96-well flat-bottomed plates with EL-4 cells (1 × 10^4^ cells/well) as previously described (Loke et al. 2000). Following a 48-h incubation, 1 μCi of [^3^H]TdR in 10 μl complete medium was added to each well, and plates were incubated overnight before harvesting and counting using a liquid scintillation counter (Microbeta 1450, Trilux). Quadruplicate measurements per sample were performed. Results were plotted in counts per minute (cpm).

### Quantification of nitric oxide (NO) and arginase activity

NO production was assessed by nitrite accumulation in the culture media using the Greiss Reagent. In brief, 100 μl culture supernatant was mixed with 100 μl of 5.8% phosphoric acid, 1% sulphanilamide, 0.1% N-(1-naphthyl) ethylenediamine dihydrochloride. Absorbance was measured at 540 nm using a microplate reader. Concentration was determined according to a standard curve of sodium nitrite solution. Arginase activity was measured according to previously published protocols (Munder et al. 1998). Briefly, 1–2 × 10^5^ cells were lysed with 100 μl 0.1% Triton X-100. Following a 30 min incubation with shaking, 100 μl of 25 mM TrisHCL and 20 μl of 10 mM MnCl_2_ were added and the enzyme activated by heating to 56 °C for 10 min. l-Arginine hydrolysis was carried out by incubating 100 μl of this lysate with 100 μl of 0.5 M l-arginine (pH 9.7) at 37 °C for 60 min. The reaction was then stopped with 800 μl H_2_SO_4_ (96%)/H_3_PO_4_ (85%)/H_2_O (1/3/7, v/v/v), and 40 μl of 9% isonitroso-propiophenone added, followed by heating to 99 °C for 30 min before reading on the microplate reader at 540 nm. A standard curve of urea solution was used to determine urea concentrations, as a readout of arginase enzyme activity. Unless otherwise stated, all reagents were obtained from Sigma–Aldrich.

### RNA extraction and real-time RT PCR

RNA was recovered from cells by re-suspension in TRizol reagent (Invitrogen). Total RNA was extracted according to the manufacturer's instructions. Following DNAse1 treatment (Ambion) to remove contaminating genomic DNA, approximately 1 μg of RNA was used for the synthesis of cDNA using MMLV reverse transcriptase (Stratagene). Relative quantification of the genes of interest was measured by real-time PCR, using the Roche LightCycler. For each gene, five serial 1:4 dilutions of a positive control sample of cDNA (macrophages elicited at peak Th2 activation from *B. malayi* implanted mice) were used as a standard curve in each reaction and the expression levels were estimated from the curve. Amplification was quantified and normalised using β-actin as a housekeeping gene. PCR amplifications were performed in 10 μl, containing 1 μl cDNA, 4 mM MgCl_2_, 0.3 mM primers and the LightCycler-DNA SYBR Green I mix (Roche). The amplification of *β-Actin*, *RELM-α* and *Arginase1* was performed as previously described (Nair et al. 2005).

### Western blotting

17 μl of the initial 1 ml peritoneal wash was mixed with sample buffer supplemented with denaturing buffer (NuPage, Invitrogen), heat denatured and resolved by SDS-PAGE using 4–12% gradient Bis–Tris gels (NuPage, Invitrogen) followed by transfer onto nitrocellulose membrane (Bio-Rad). The blot was blocked for 30 min in Pierce StartingBlock. Primary Abs were diluted in Pierce StartingBlock + 0.05% Tween-20: Anti-Ym1 (Nair et al. 2005) and Anti-RELMα (Peprotech) and incubated with the blots overnight at 4 °C. Incubation with goat-anti-rabbit HRP: 1/2000 for 1 h was followed with detection by enhanced chemiluminescence method according to the manufacturers instructions (ECL kit; Amersham). Signal produced was detected using film (*Hyperfilm*: Amersham ECL Hyperfilm) and MultiImage light cabinet along with the Fluorchem programme (Alpha Innotech) were used to measure relative protein concentrations on each blot.

### Counting of microfilaria

10 μl PECs were added to 200 μl FACS lysing solution (BD-Biosciences) to fix microfilariae. Following centrifugation for 5 min at 3000 × *g* and removal of the supernatant, cells/microfilaria were briefly resuspended and all the microfilariae in each sample were counted by microscopic examination.

### *In vitro* splenocyte cultures

Spleens were removed and single cell suspensions prepared. These were cultured in 96-well round bottom plates at 1 × 10^6^ cells/well containing either 10 μg/ml parasite extract (BmA) or 1 μg/ml Concanavalin A (ConA) or medium alone (complete RPMI) at 37 °C. After 72 h culture, supernatants were removed for cytokine assay. BmA was prepared as previously described, by homogenisation of mixed adult nematodes in PBS (Tawill et al. 2004).

### Cytokine assay

IL-4, IL-5, IL-13, IFNγ and IL-10 in culture supernatants were measured using BD Cytometric Bead Array Flex sets. Samples were acquired on FACSArray analyser (BD Biosciences) and the amount of cytokine present calculated using FCAP analysis software (BD Biosciences).

### Data analysis

Graphs were prepared using PRISM (GraphPad software, Berkeley, CA). The Mann–Whitney test was used to test for significance as indicated in the figure legends.

## Results

### WT and MyD88^−/−^ BMMφ alternatively activate in response to IL-4 *in vitro*

To determine whether MyD88 is necessary for the alternative activation of macrophages, we compared the ability of BMMφ cultured from WT and MyD88^−/−^ to respond to IL-4 *in vitro*. Both Arginase 1 and iNOS activity were measured to represent the competing arms of the arginine metabolism pathway associated with alternative *vs.* classical macrophage activation, respectively (Munder et al. 1998). To further characterise the macrophage phenotype, we assessed the mRNA expression of *Arginase 1*, *RELM-α* and *Ym-1*, as accepted markers of alternative activation (Jenkins and Allen 2010).

**Fig. 1:**
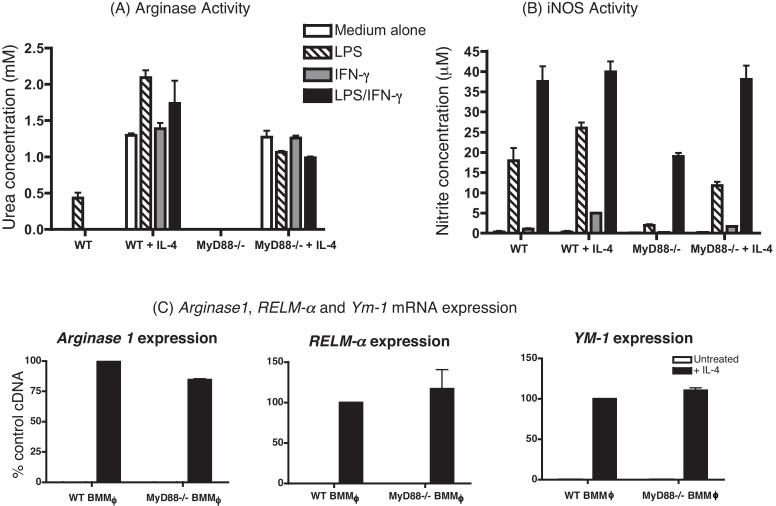
MyD88 deficiency had no effect on the alternative activation of Mφ *in vitro*. BMMϕ were preteated o/n with IL-4 and then stimulated with LPS and IFN-γ together or separately for 16–20 h. Urea concentration is shown as a measure of arginase activity (a) and nitrite as a measure of iNOS activity (b). mRNA was extracted and realtime RT-PCR for *Arginase 1*, *RELM-α* and *YM-1* expression carried out. Black bars here represent IL-4 treatment (c). mRNA expression is shown as a % of a positive control sample and was normalised to β-actin. Results are shown as the mean of replicate samples (±SEM) and are representative of three experiments.

Macrophages were cultured with or without IL-4 overnight before treatment with LPS and IFN-γ, either together or separately, or with medium alone, for 20–24 h. After this time, arginase and iNOS enzyme activities were measured in the cell lysates and culture supernatants, respectively (Fig. 1A and B). mRNA expression of *Arginase 1*, RELM*-α* and *Ym-1* was measured in the harvested cells (Fig. 1C). Both WT and MyD88^−/−^ macrophages up-regulated arginase activity in response to IL-4 (Fig. 1A). As previously reported LPS also stimulated WT macrophages to produce arginase (Louis et al. 1998) and, as expected, this response was abolished in MyD88^−/−^ mice. There was no impairment in the ability of MyD88^−/−^ BMMφ treated with IL-4 to produce other markers of alternative activation, including *RELM-α* and *Ym-1* (Fig. 1C). Thus, there is no apparent deficiency in the fundamental ability of macrophages to become alternatively activated in MyD88^−/−^ animals.

Nitrite in the supernatants of the cultured macrophages was assessed using the Greiss reagent, as a measure of iNOS activity. As expected, WT BMMφ produced nitrite when treated with LPS alone, and with IFN-γ. The two stimuli together had a synergistic effect on iNOS activity (additive with IL-4 pre-treatment; Fig. 1B). MyD88^−/−^ BMMφ also produced NO synergistically when treated with LPS and IFN-γ together but could not produce NO upon treatment with LPS alone, except following pre-treatment with IL-4.

### Th2 cytokine production is not significantly altered in *B. malayi*-implanted mice in the absence of MyD88

**Fig. 2:**
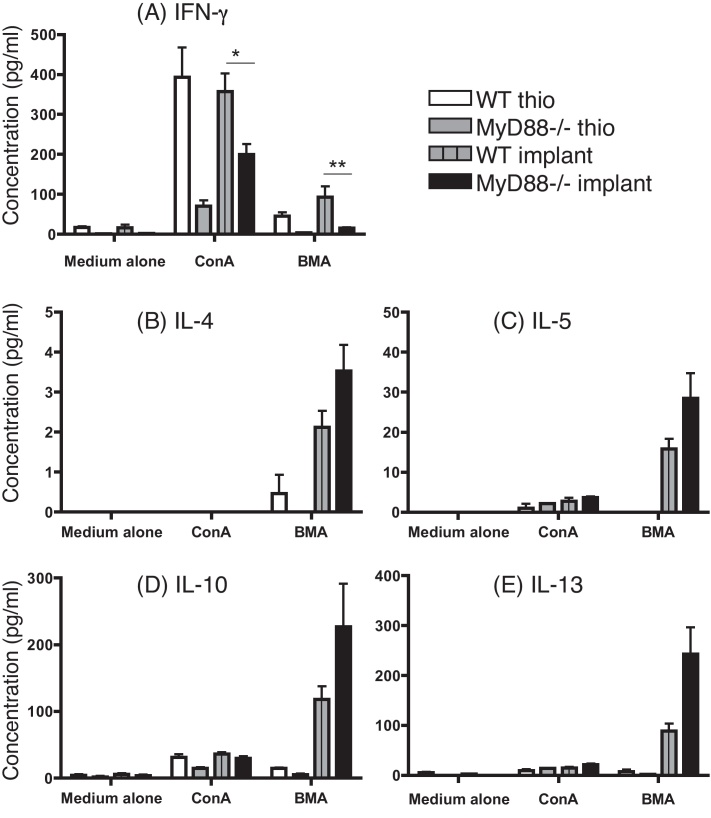
The Th1 response is impaired in MyD88^−/−^ mice but the Th2 response is not significantly altered in *B. malayi*-implanted animals. Splenocytes were recovered from C57BL/6 mice 19 days after *B. malayi* implant or 3 days after injection with thioglycollate (thio). Splenocytes were treated with media alone, Concanavalin A (ConA) or *B. malayi* antigen (BMA) for 72 h before the supernatants were removed and levels of IFN-γ (a), IL-4 (b), IL-5 (c), IL-10 (d) and IL-13 (e) measured by cytometric bead array. Significant differences were determined by the Mann–Whitney test *p < 0.05, **p < 0.01. These results are representative of three experiments.

Before determining the impact of MyD88 deficiency on macrophage activation status *in vivo*, it was important to first ascertain if there would be any impairment or enhancement in the overall Th2 response in *B. malayi* implanted mice. For this, the Th2 cytokines IL-4, IL-5, IL-10 and IL-13, as well as IFN-γ as a marker of Th1 activation, were measured from the supernatants of cultured splenocytes treated with medium alone, ConA or BmA (Fig. 2A–E). As expected, all Th2 cytokines were increased in an Ag-specific manner in response to BmA in WT implanted mice. Ag-specific production of the Th2 cytokines IL-4, 5, 10 and 13 was further elevated in the MyD88^−/−^ implanted mice, but this did not reach statistical significance (measured by Mann–Whitney; Fig. 2B–E). In agreement with previous reports, the Th1 response was significantly impaired in MyD88^−/−^ animals, as measured by IFN-γ production by cultured splenocytes (Fig. 2A). This was true for both parasite implanted and thioglycollate injected MyD88^−/−^ mice, compared to their WT counterparts. The difference in IFN-γ production between WT and MyD88^−/−^ implanted mice was found to be statistically significant in response to both ConA and BmA. This trend was also seen between the WT and MyD88^−/−^ thioglycollate-treated mice. Overall, these results show that the Th2 response is not impaired in MyD88^−/−^ mice implanted with *B. malayi*.

### The absence of MyD88 does not affect cellular accumulation after *B. malayi* implant or thioglycollate injection

**Fig. 3:**
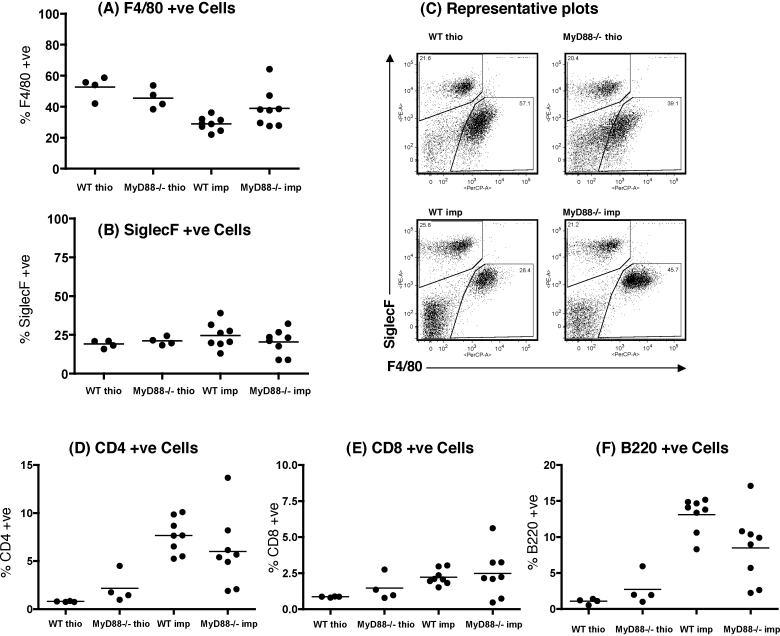
Similar numbers of Mφ, eosinophils, and lymphocytes, are found in wild type (WT) and MyD88^−/−^ mice. 19 days after implant (3 days after thioglycollate injection; thio) PECs from mice on the C57BL/6 background were recovered and double-stained for F4/80 (a) and SiglecF (b). Sample plots are shown in (c). PECs were also stained for CD4 (d), CD8 (e) and B220 (f). Results are representative of three experiments.

PECs were recovered from WT and MyD88^−/−^ mice surgically implanted with *B. malayi* adult worms (d19) or injected i.p. with thioglycollate for three days. All animals exhibited large increases in cell number (5–20 × 10^6^ PECs/mouse) but there was no significant difference in total cell numbers between the four experimental groups. As reported previously macrophages, eosinophils and lymphocytes make up the majority of cells in the peritoneal cavity of *B. malayi* implanted mice (MacDonald et al. 2003). To address whether a lack of MyD88 affected the cellular profile in response to these stimuli, we examined the proportions of F4/80 + macrophages, SiglecF + eosinophils as well as lymphocyte subsets (Fig. 3) present in the PEC by flow cytometry. No significant differences in the proportion of macrophages or eosinophils were observed between WT and MyD88^−/−^ animals (Fig. 3A and B). This was confirmed by microscopic examination of cytocentrifuge preparations for macrophages, eosinophils and lymphocytes (data not shown). Flow cytometric analysis of lymphocyte subsets showed equivalent proportions of CD4 + and CD8 + T cells in the PECs of both WT and MyD88^−/−^ implanted mice (Fig. 3D and E). B220 was used as a marker of B cells and although there appear to be fewer B cells in implanted MyD88^−/−^ animals, this was not significant (Fig. 3F).

### Absence of MyD88 does not affect arginase production or suppressive ability of macrophages generated following *B. malayi* implant

**Fig. 4:**
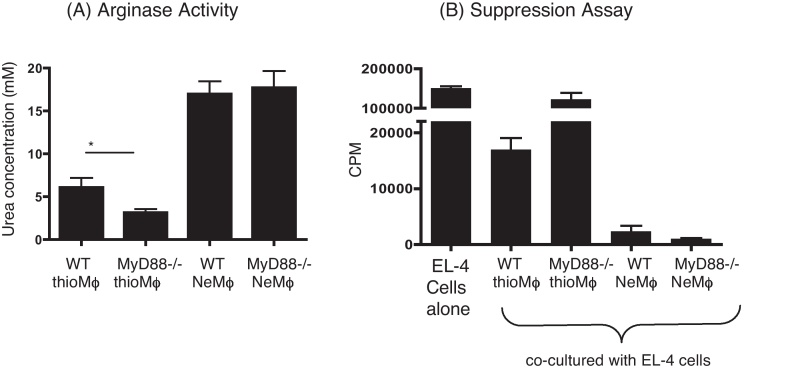
No difference in the arginase activity or suppressive ability of NeMϕ in WT or MyD88^−/−^ mice after implantation with *B. malayi* worms. PECs were recovered from C57BL/6 mice 19 days after *B. malayi* implant or 3 days after injection with thioglycollate (thio). Macrophages were purified by adherence and arginase enzyme activity calculated (a). Suppressive ability was measured by replacement of the medium and co-culture with EL-4 thymoma cells. After 48 h, the EL-4 cell proliferation was assessed by [^3^H] thymidine incorporation (b). Significant differences were determined by the Mann–Whitney test *p < 0.05. These results are representative of three experiments.

To assess macrophage phenotype following nematode implant, PECs were purified by adherence, and the levels of arginase activity measured (Fig. 4A). As expected, nematode elicited macrophages (NeMφ) produced more arginase than thioglycollate elicted macrophages (ThioMφ). However, there was no significant difference in the levels of arginase produced when comparing WT and MyD88^−/−^ NeMφ. Notably, there was a small but significant reduction in arginase activity in MyD88^−/−^ relative to WT ThioMφ (Fig. 4A).

We assessed proliferative suppression *ex vivo* by NeMφ (Loke et al. 2000; Mylonas et al. 2009) to investigate whether the absence of MyD88 would influence this feature of alternative activation. As expected, responder (EL-4) cell proliferation was reduced on co-culture with NeMφ, in comparison to control ThioMφ, and this was still the case for NeMφ generated in MyD88^−/−^ animals. We have previously observed that ThioMφ are also able to inhibit proliferation of co-cultured cells (Mylonas et al. 2009) but unlike NeMφ-mediated suppression this occurs in an IL-4 independent manner (unpublished). Here we demonstrate that the suppressive ability of WT ThioMφ is entirely dependent on MyD88 (Fig. 4B).

### Absence of MyD88 does not affect microfilarial numbers, or expression of the alternative activation markers Ym1 or RELM-α, in the peritoneal fluid of implanted mice

**Fig. 5:**
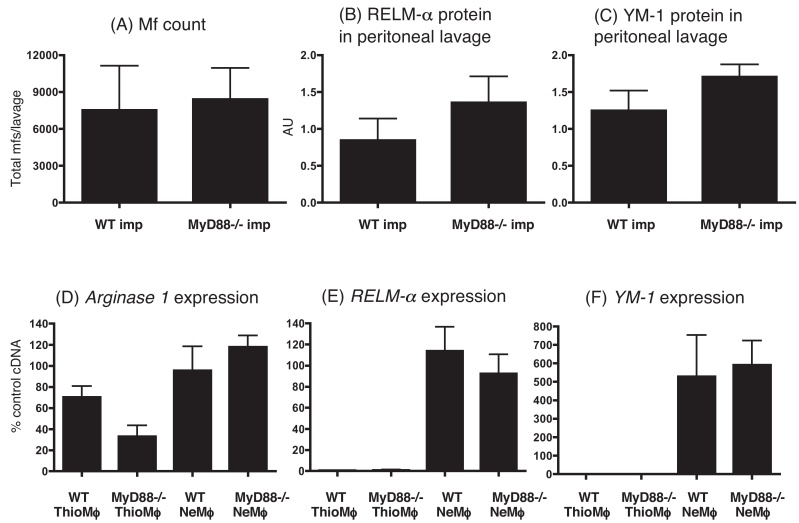
The absence of MyD88 had no effect on microfilaria numbers or alternative activation markers in the peritoneal cavity after *B. malayi* implant. At day 19 after *B. malayi* implant of C57BL/6 mice, i.p. lavages were carried out and numbers of microfilaria present enumerated (a). The lavage fluid was tested for RELM-α (b) and YM-1 (c) by Western blot. Macrophages were purified by adherence from peritoneal lavages and RNA extraction and realtime RT-PCR was carried out for *Arginase1* (d), *RELM-α* (e) and *YM-1* (f). mRNA expression is shown as a % of a positive control sample and was normalised to β-actin. These results are representative of three experiments.

We next wanted to address whether absence of MyD88 affected worm viability. Assessment of the first larval stage of a *B. malayi*, the microfilariae produced by the implanted female worms, provides an indication of worm viability (Rao and Well 2002). After 19 days of *B. malayi* infection, the peritoneal lavage fluid was extracted and the numbers of microfilariae were counted from both WT and MyD88^−/−^ animals (Fig. 5A). No difference was detected, suggesting that MyD88 deficiency had neither a positive nor negative effect on worm survival and fecundity in this model of filarial nematode infection.

Since the alternative activation markers Ym1 and RELM-α are secreted proteins, Western blots were carried out in order to measure the levels of these mediators in the peritoneal lavage fluid of nematode-implanted mice (Fig. 5B and C). Once again no significant difference was found between the WT and MyD88^−/−^ animals. However, there was a trend towards higher RELM-α expressed by the MyD88^−/−^ mice (Fig. 5B), consistent with the trend towards slightly higher Th2 induction seen in these experiments (Fig. 2B–E).

RNA was also extracted from purified peritoneal macrophages and quantitative RT-PCR carried out to assess levels of *Arginase1*, *RELM-α* and *Ym-1* mRNA expression (Fig. 5D–F) to see whether these levels would correlate with Ym-1 and RELM-α protein production (Fig. 5B and C) and arginase activity (Fig. 4A). Measurements of *Arginase1*, *RELM-α* and *Ym-1* (Fig. 5D–F) mRNA showed a close correlation between mRNA and protein expression for these alternative activation markers, as previously observed (Nair et al. 2005).

## Discussion

We have shown that macrophages isolated from a chronic *in vivo* Th2 setting do not require the adaptor protein MyD88 for the induction of alternative activation markers. Additionally, we found no impairment in the Ag specific Th2 response to *B. malayi* in the absence of MyD88 (Fig. 2). Previous studies of microbial infection using MyD88^−/−^ mice have shown evidence of significantly enhanced Th2 responses (Muraille et al. 2003; Chen et al. 2010; Gaddis et al. 2011). MyD88^−/−^ mice infected with the gastrointestinal nematode *Trichuris muris* also display elevated Th2 responses relative to their WT counterparts resulting in enhanced resistance to infection (Helmby and Grencis 2003). This augmentation of type 2 cytokines is far greater than the trend we observed in our current study. This is likely explained by the fact that *T. muris* worms burrow within the cecal epithelium, exposing these cells to commensal bacteria (Cliffe and Grencis 2004), which would act as a powerful stimulus of the Th1 response *via* MyD88-dependent pathways. The increase in the Th2 response in MyD88^−/−^ animals infected with *T. muris* can thus be explained by an inability to mount an effective Th1 response against the bacteria to which they are exposed (deSchoolmeester et al. 2006). This is supported by reports showing that, in the absence of MyD88, MyD88-independent signaling through TLR4 can confer the ability to support Th2 responses (Kaisho et al. 2002).

Relative to *T. muris*, the peritoneal environment of the *B. malayi* implant model is essentially ‘sterile’, with no commensal bacteria. Thus the limited impact of MyD88 deficiency on Th2 immunity may not be surprising. However, as previously mentioned, *B. malayi* contains endosymbiotic bacteria which might be expected to influence the immune response (Turner et al. 2009). Nonetheless, despite strong *in vitro* evidence that *Wolbachia* ligands can signal through TLRs in a MyD88-dependent fashion (Hise et al. 2007) we saw little effect of MyD88 deficiency on Th2 or AAMφ development. In this implant model, live adult *female B. malayi* produce large numbers of larval offspring and some microfilarial death would be expected across the 19 day period, which should expose the host to *Wolbachia*. Our data thus suggest that in the context of live filarial nematode infection, the influence of *Wolbachia* on the host response may not be as great as previously presumed. This does not diminish, and indeed may enhance, the role of *Wolbachia* as target for filarial chemotherapy (Hoerauf 2008). The lack of a significant increase in the Th2 response in the absence of MyD88 in our work would be consistent with studies showing no role for MyD88 in dendritic cell (DC) induction of Th2 cells against helminth Ag (Kane et al. 2008). A limited role for MyD88 would also support the importance of DCs rather than basophils, in helminth induced Th2 responses (Ohnmacht and Voehringer 2009; Kim et al. 2010; Phythian-Adams et al. 2010), as MyD88 is implicated in the ability of basophils to produce IL-4 (Kroeger et al. 2009).

Although we found no significant difference in the magnitude or character of the T cell response between WT and MyD88^−/−^ implanted mice, MyD88 deficiency could still have had cell intrinsic effects on the ability of Mφ to respond to *in vivo* signals. However, this did not seem to be the case as MyD88 deficiency had no effect on the ability of NeMφ to suppress the proliferation of co-cultured EL-4 cells or on the expression of any alternative activation markers that we assessed. Not surprisingly, the absence of any change in effector cell function or numbers translated into no effect on *B. malayi* worm viability, as we found similar numbers of microfilariae in the peritoneal cavity of both WT and MyD88^−/−^ animals. Significantly, we also found no impact on macrophage activation state or Th2 response in *B. malayi* implanted C3H/HeJ mice, which cannot signal through TLR4 (unpublished report from the Marine Biological Laboratory Biology of Parasitism course – 2006). Thus, MyD88-independent TLR4 signaling is unlikely to contribute to the NeMφ phenotype.

Although MyD88 deficiency had a limited impact on parasite-implanted mice, it did influence the response to thioglycollate treatment. This indicates that a TLR stimulus may be at least partially required for cell recruitment and macrophage phenotype induced by thioglycollate. There was a decrease in arginase activity in MyD88-deficient ThioMφ (Fig. 4A) and also a decrease in the suppressive ability of these macrophages compared to WT (Fig. 4B). These facts are likely to be linked, as arginase can play a major role in macrophage-mediated suppression (Pesce et al. 2009). However, this effect of arginase may reflect only part of the story, given the relatively large difference in suppressive ability of WT ThioMφ, in relation to MyD88^−/−^ ThioMφ (WT approx. 10× more suppressive; Fig. 4B), compared to the differences in arginase enzyme activity (WT display approx. 2× more arginase activity than MyD88^−/−^; Fig. 4A).

Our *in vitro* work using BMMφ provided direct evidence that there is no deficiency in the ability of IL-4 to generate AAMφ in the absence of MyD88. In agreement with Louis et al. 1998, *in vitro* treatment of WT BMMφ with LPS increased arginase production (Fig. 1A, left). The complete absence of arginase activity in LPS-treated MyD88^−/−^ BMMφ are consistent with findings highlighting the importance of TLR-mediated arginase production by microbial pathogens (Kasmi et al. 2008). Another interesting aspect of the *in vitro* studies was the finding that while WT BMMφ produced nitric oxide in response to LPS, the response in MyD88^−/−^ BMMφ was minimal unless LPS was combined with IFN-γ (Fig. 1B). A possible interpretation of these results is that, in the absence of MyD88, LPS signals through TLR4 *via* a MyD88-independent pathway, for example through interferon regulatory factor (IRF)-3, which causes the upregulation of IFN-β, but not iNOS. IFN-β might in turn upregulate the transcription factor IRF-1 (Fujita et al. 1989) which, when coupled with IFN-γ, would lead to the production of NO. This hypothesis is supported by previous reports showing that LPS augmentation of *iNOS* mRNA expression by IFN-γ is due to IRF-1 upregulation by LPS (Koide et al. 2007).

In summary, we have found using both *in vivo* and *in vitro* approaches that MyD88 signaling is not an essential requirement for alternative activation of Mφ. Further, we have shown that in the context of a Wolbachia-containing filarial nematode, MyD88 does not significantly contribute to the overall character of the immune response. Importantly, we are not ruling out the contribution of TLRs to immune profiles in other nematode infection settings. Indeed, TLR activation by helminth molecules has important known roles in the modulation of innate immunity (reviewed in Perrigoue et al. 2008) and as discussed above, TLR-signaling influences the response to gut dwelling nematodes. We would further expect that helminths involved in tissue migration and damage would trigger damage associated molecular pattern molecules (DAMPs) that bind TLRs (Liu et al. 2012). This is particular relevant to evolutionary associations of Th2 immunity with wound repair (Allen and Wynn 2011). Importantly, this study has not addressed the contribution of many other PRRs such as the NOD-like or C-type lectin receptors to Th2 activation or alternative macrophage activation. In particular, *Schistosoma mansoni* and *Toxocara canis* glycans have both been demonstrated to have parasite-specific ligands that bind the C-type lectin, DC-SIGN, with potential for the modulation of dendritic cell responses (Meyer et al. 2005; Schabussova et al. 2007). Indeed, such interactions may act co-operatively with TLR ligands to modulate the host immune response as shown for *S. mansoni* glycolipids (van Stijn et al. 2010). Thus, despite our finding that the absence of TLR signaling does not significantly alter the host response to *B. malayi*, the interaction of parasite-specific ligands with host innate receptors remains a fruitful area of investigation.
